# Ki67 proliferation index augments two-tier tumour grade in prediction of survival and progression-free survival in epithelioid pleural mesothelioma

**DOI:** 10.1183/23120541.01300-2025

**Published:** 2026-06-08

**Authors:** Geraldine A. Lynch, Yu Zhi Zhang, Nidhi Bhatt, Pia Charters, Avinash Aujayeb, Saru Walker, Jenny Symonds, Amelia O. Clive, Rahul Bhatnagar, Anna Morley, Paul White, Andrew G. Nicholson, Nick A. Maskell, Anna Bibby

**Affiliations:** 1 Academic Respiratory Unit, Southmead Hospital, University of Bristol, Bristol, UK; 2 Southmead Hospital, North Bristol NHS Trust, Bristol, UK; 3 Royal Brompton and Harefield Hospitals, Guy's and St Thomas’ NHS Foundation Trust, London, UK; 4 Royal United Hospitals Bath NHS Foundation Trust, Bath, UK; 5 Northumbria Healthcare NHS Foundation Trust, Newcastle Upon Tyne, UK; 6 Department of Mathematics and Statistics, University of West of England, Bristol, UK; 7 A list of the members of the ASSESS-Meso Collaborative Group is in the Acknowledgements

## Abstract

**Background:**

Pleural mesothelioma (PM) is a fatal asbestos-related cancer with a poor and often uncertain prognosis. This study validates the histological proliferation marker Ki67 and evaluates whether its integration into the two-tier tumour grading system can improve prognostication in epithelioid PM.

**Methods:**

Patients with epithelioid PM were recruited from two longitudinal cohort studies from 2010–2023. Diagnostic biopsies were analysed by three pulmonary pathologists. Cox regression determined the relationship between covariables and outcomes. Pearson correlation assessed the association between Ki67 and two-tier grade. A prognostic model combining Ki67 and tumour grade was internally validated using bootstrapping.

**Results:**

98 patients were recruited. Ki67 was strongly predictive of overall survival (OS) and progression-free survival (PFS) and correlated with two-tier tumour grade. 30% was the optimal cut-off, with Ki67 more strongly predictive of OS (hazard ratio (HR) 2.37, 95% CI 1.51–3.71) and PFS (HR 2.09, 1.35–3.23) than two-tier grade (HR 1.83, 1.13–2.97 and HR 1.70, 1.08–2.66, respectively). Combining Ki67 and two-tier grade improved prediction of OS and PFS compared with two-tier grade alone. Ki67 stratified patients within each tumour grade, with median survival in the lowest risk group (low Ki67, low grade) of 660.5 days (IQR 329–1297) and 300 days (IQR 124–366) in the highest risk group (high Ki67, high grade).

**Conclusion:**

Ki67 is a valid surrogate for tumour grade with an optimal cut-off at 30%. Integrating Ki67 into the two-tier grading system enhances prognostic accuracy, improves outcome prediction and would reduce uncertainty for patients and clinicians.

## Shareable abstract

**Adding Ki67 to tumour grade outperforms tumour grade alone for prognostication in epithelioid PM. Ki67 effectively risk stratifies within each tumour grade. Tumour grade plus Ki67 should be considered as a new reporting standard in epithelioid PM.** https://bit.ly/437RtRP

## Background

Pleural mesothelioma (PM) is a primary malignancy of the lung lining caused by asbestos exposure. Despite recent treatment advances, prognosis remains poor. The 3-year survival has shown little improvement, from 7% to 10% in recent years [1]. Predicting prognosis is important for patients and their families in planning for the future [2], particularly given variable treatment response rates [3]. Many patients are either unable to receive treatment or choose to monitor their disease before making treatment decisions [4] and are increasingly reliant on prognostic information provided at diagnosis.

Factors associated with poorer prognosis include male sex, older age, poorer performance status (PS) and non-epithelioid histology [5–7]. Several biomarkers have been evaluated for prognostication but none are recommended in current guidelines [8–10].

While epithelioid mesothelioma carries the best prognosis, it is a heterogenous subtype with variable survival. The two-tier tumour grading system was introduced into the 2021 World Health Organisation (WHO) classification to improve prognostic discrimination. Its reporting is recommended by the International Collaboration on Cancer Reporting (ICCR) [11, 12]. A large validation study by Zhang *et al.* [13] showed that median survival of people with low grade disease was more than double that of those with high-grade disease.

Galeano *et al.* [14] also validated tumour grade and investigated Ki67, a proliferation marker currently used to guide treatment decisions in peritoneal mesothelioma [15]. They showed that Ki67 correlated with tumour grade, suggesting that it could be used as a surrogate.

This study aimed to assess the value of Ki67 in PM prognostication and evaluate its addition to the tumour grading system. We assessed the reproducibility of two- and three-tier nuclear grade and Ki67 between raters. With a wide range of Ki67 cut-offs reported in the literature [16, 17], we evaluated the optimal dichotomisation point. Finally, we compared the prognostic ability of Ki67 and tumour grade with other established histological markers and prognostic scoring systems.

## Methods

This manuscript complies with the TRIPOD guidelines and the STROBE statement for cohort studies [18, 19]. Patients with PM and their carers were involved in formulating the study objectives and interpreting results *via* patient and public involvement engagement sessions.

### Patient selection

Patients with epithelioid PM were identified from two prospective UK cohort studies approved by the regional Southwest Ethics committee: the Pleural Investigation Study (08/H0102/11) and ASSESS-Meso (17/SW/0019) [20], over a 13-year period from January 2010 to November 2023.

Eligibility criteria included: age over 18 years, histologically confirmed PM and ability to give written informed consent. Cases from two centres (Bristol and Northumbria) were included for geographical variation.

Sample size was determined pragmatically by biopsy availability. All patients were treatment naive at the time of biopsy. Albumin, and serum neutrophil and lymphocyte count at diagnosis were recorded, with neutrophil–lymphocyte ratio (NLR) calculated. Survival data, treatment status and disease progression were recorded. Progression was defined as radiological evidence of progression with CT interval clinically determined; clinical deterioration in the absence of radiological progression was not recorded.

### Histological analysis

Diagnostic biopsies were retrieved from NHS pathology laboratories as formalin-fixed paraffin-embedded blocks. All cases were stained with haematoxylin and eosin for diagnosis confirmation and assessment of tumour grade and for BRCA-1-associated protein-1 (BAP1) and Ki67 immunohistochemistry (IHC).Technical details of the Ki67 staining protocol can be found in the supplementary material (appendix A).

Cases were independently reviewed by three thoracic pathologists blinded to survival. Cases were included if deemed epithelioid subtype by at least two pathologists.

Cases were evaluated for the degree of nuclear atypia and mitotic activity and necrosis. Mitotic count and nuclear atypia were translated into scores based on the WHO tumour grading system with three-tier and tumour grade calculated [11].

The degree of host response and the presence of tertiary lymphoid structures (TLSs) were recorded. Host response was graded mild, moderate or severe (≤10%, 11% to ≤50%, and >50% inflammatory tissue, respectively). Architectural patterns and stromal features were recorded. The presence or absence of BAP1 staining was assessed, with BAP1 loss defined as the loss of nuclear BAP1 staining in all tumour cells.

Where pathologists disagreed, the majority opinion was used. In the absence of majority consensus, the highest reading was taken.

For Ki67 assessment, the area of maximum intensity was chosen by scanning at low power and confirmed at medium power ×100). The percentage of positive tumour cells was calculated at high power view (×400) on >500 tumour cells. Tumour cells staining positive for Ki67 were counted and calculated as a proportion of the total number of tumour cells within the hotspot in the high-powered view to calculate Ki67%. Consensus Ki67 was calculated as the average of the individual scores.

### Statistical analysis

The primary outcome was overall survival (OS), defined as time from diagnosis to death, censored on 16 August 2024. Secondary outcome was progression-free survival (PFS), recorded as time between diagnosis and radiological disease progression as reported by a thoracic radiologist or death; whichever occurred first. Explanatory variables were Ki67 (alone and in combination with tumour grade) and the pre-specified markers albumin, NLR, host response, nuclear atypia, mitotic activity, necrosis, TLS and two- and three-tier nuclear grade.

Interobserver variation was evaluated using the average Cohen's kappa for pairs of raters, compared with Fleiss's kappa for three raters. A weighted kappa was used for ordinal variables with more than two categories. Agreement was categorised as per Landis and Koch's (<0 poor, 0–0.2 slight, 0.21–0.4 fair, 0.41–0.6 moderate, 0.61–0.8 substantial and 0.81–1.0 as almost perfect) [21]. Bland–Altman plots were created for each observer pair to visualise measurement agreement.

Descriptive statistics summarised patient characteristics and baseline data. Univariable Cox regression was used to determine the relationship between the explanatory variables and outcomes, with multivariable Cox regression used to adjust for pre-specified potential confounders of age, sex and WHO PS. Missing data were handled by complete–case analysis if proportion of missing values was minimal. The optimal Ki67 cut-off was explored using univariable Cox regression on quartiles derived from the data, as well as the following cut-offs, which have been used in previous literature:15%, 22% [16] and 30% [14, 22]. For each candidate threshold, hazard ratios (HRs) and overall model fit (log-likelihood) were calculated and compared to balance the statistically optimal threshold with a clinically reproducible choice. We used a chi-squared test to assess the associations between covariables and Ki67.

Multivariable Cox regression was used to combine Ki67 with tumour grade, with Ki67 as continuous and binary variables unadjusted and adjusted for confounders. Proportional hazards and linearity assumptions were tested. Ki-67 was modelled as a continuous variable using restricted cubic splines with six knots placed at selected percentiles of the observed distribution. Outliers and interaction terms were explored. Harrell's c-statistic tested discriminative performance. Internal validation using 500 bootstrapped samples was performed to estimate optimism in calibration slope and c-statistic.

Dichotomised Ki67 and two-tier grade were separated into four groups; Cox regression and Kaplan–Meier curves assessed OS and PFS. The same analysis was performed for low and high tumour grade subgroups.

Sensitivity analysis compared Ki67, tumour grade and the clinically chosen model (two-tier grade and Ki67 combined) based on treatment status using univariable and multivariable Cox regression.

Exploratory analysis compared the clinically chosen model with models combining Ki67 with individual components of the tumour grade, or all components of tumour grade without mitosis. Harrell's c-statistic and likelihood ratio tests were used to compare models.

We used Stata v.18 (StataCorp, 2023) for the analyses.

## Results

**FIGURE 1:**
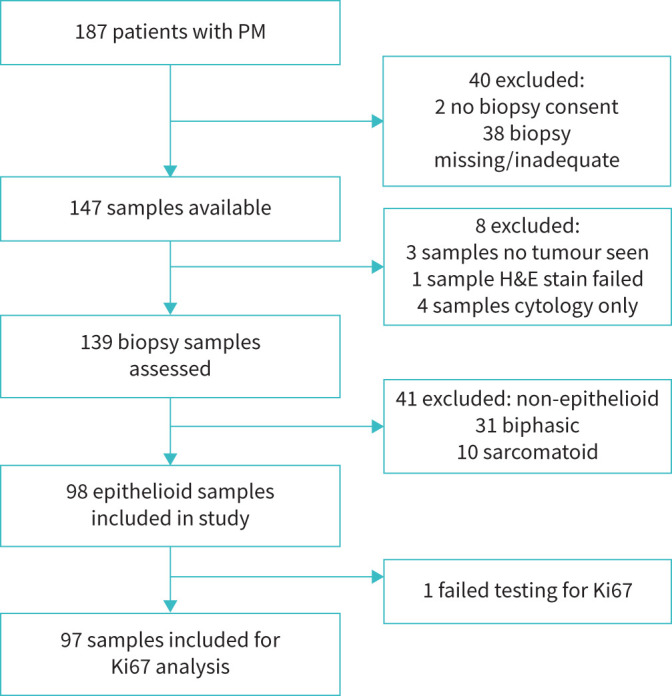
CONSORT diagram for the study. PM: pleural mesothelioma; H&E: haematoxylin and eosin.

187 cases of PM were identified. 139 had biopsy samples suitable for assessment, with 98 confirmed as epithelioid subtype (46 from the pleural investigations cohort, 52 from ASSESS-Meso) (figure 1). One sample failed IHC staining for Ki67 and was excluded from Ki67 analyses.

The minimum follow-up was 9 months. Of the 98 participants, 83 died during follow-up. The remaining 16 patients were censored on 16 August 2024 after a median follow-up of 759 days (IQR 497–1291).

Baseline characteristics are shown in table 1. 82.7% of patients were men and median age was 74.5 (IQR 70.5–79.7). WHO PS was missing in 4 of 98 (3.1%) and tumour stage in 8 of 98 (7.1%). 86 people had treatment information recorded (supplementary material, appendix B), of whom 47 (54.6%) received active anticancer treatment. 38 (44.1%) participants received single modality treatment, usually systemic therapy (31 of 86 (36.0%) chemotherapy, 5 of 86 (5.8%) immunotherapy). Seven people (8.1%) underwent dual modality treatment, of whom six received chemotherapy and immunotherapy and one received chemotherapy with radiotherapy. Two patients (2.3%) received trimodality treatment in the form of chemotherapy followed by extended pleurectomy/decortication surgery and immunotherapy, as part of the MARS2 trial [23].

**TABLE 1:** Baseline characteristics

Baseline characteristic	Total n	Result
**Age, days**	98	74.5 (70.5–79.7)
**Male**	98	81 (82.7)
**PS 0–1**	94	80 (82)
**Received active anticancer treatment**	86	47 (54.6)
**Tumour stage IA-II (TNM8)**	90	60 (66.7)
**Ki67%**	97	24.3 (13.0–45.3)
**Ki67≥30%**	97	41 (42.2%)
**NLR**	95	4.1 (2.7–6.3)
**Albumin, g·L^−1^**	90	34.5 (31–37)
**Overall survival, days**	98	385.5 (221–780)
**Progression-free survival, days**	98	181 (106–376)

Data are presented as n (%) or median (interquartile range). PS: performance status; NLR: neutrophil–lymphocyte ratio.

### Interobserver agreement

Fleiss’ and Cohen's kappa values were comparable. There was substantial agreement between raters in tumour grade (0.67), necrosis (0.67), nuclear atypia (0.55) and Ki67 dichotomised at 30 (0.71); with almost perfect agreement in assessment of BAP1 (0.97). Agreement in TLS was moderate (0.60), with fair agreement for three-tier nuclear grade (0.35), host response (0.36) and mitotic score (0.35). These are detailed in appendix C (supplementary material). Bland–Altman analysis showed no evidence of systematic bias across the Ki–67 range. Reviewer scores were broadly consistent, though variability differed between rater pairs, with some reviewers demonstrating greater consistency than others (appendix D1–3, supplementary material).

### Primary analysis: Ki67 as continuous variable

Ki67 as a continuous variable was strongly associated with OS (crude HR 1.02, 95% CI 1.01–1.03, adjusted HR 1.02, 1.01–1.03) and PFS (crude HR 1.02, 1.01–1.02, adjusted HR 1.02, 1.01–1.03). Tumour grade was also associated with OS (crude HR 1.83, 1.13–2.97, adjusted 1.55, 0.89–2.70) and PFS (crude HR 1.70, 1.08–2.66, adjusted HR 1.66, 1.00–2.78).

### Ki67: determining the optimal cut-off

Dichotomisation of Ki67 at 28%, 29% and 30% achieved similar results (appendix E, supplementary material) with the optimal cut-off 30% based on log-likelihood, HR and clinical reproducibility. To further illustrate the continuity of risk, Ki-67 was modelled as a continuous variable using restricted cubic splines. The HR increased progressively across the range, with the steepest gradient observed between 20% and 30%, supporting the inflection point at 30% (appendix F, supplementary material).

**FIGURE 2:**
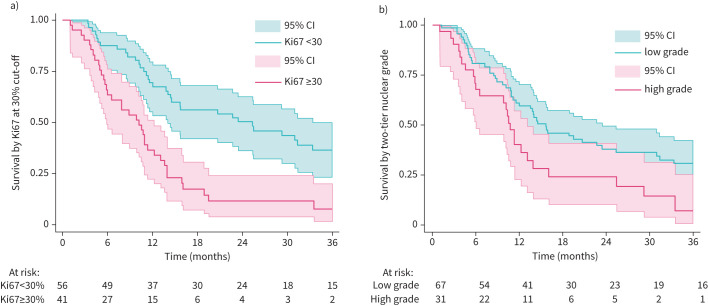
a) Kaplan–Meier survival curve for Ki67 dichotomised at 30%. b) Kaplan–Meier survival curve for tumour grade.

Ki67 dichotomised at 30% predicted OS (crude HR 2.37, 1.51–3.71, adjusted HR 2.63, 1.53–4.49) and PFS (crude HR 2.09, 1.35–3.23, adjusted HR 1.94, 1.17–3.20). Figure 2a shows Kaplan–Meier survival curve for OS with the survival curve for tumour grade shown in figure 2b (see appendix G, supplementary material for PFS curves). Median survival for people with Ki67<30% was 649.5 days (IQR 322–1263.5) and 310 days (IQR 158–416) for Ki67≥30%. Median PFS was 264 days (IQR 141–411) for Ki67<30% and 143 days (IQR 93–236) for Ki67≥30%.

### Secondary analysis: tumour grade and Ki67

The pre-specified prognostic model of tumour grade and Ki67 (analysed as a continuous variable) was predictive of OS and PFS, with a c-statistic for OS of 0.65, 95% CI 0.58–0.72 compared with 0.56, 0.50–0.62 for tumour grade alone likelihood ratio testing confirmed a significant improvement for the combined model compared with tumour grade alone (p=0.0021). The c-statistic for PFS for the combined model was 0.61, 0.55–0.67 and 0.57, 0.51–0.62 for tumour grade, with likelihood ratio testing confirming the benefit of adding Ki67 (p=0.014).

While the predictive value of Ki67% was retained for OS and PFS when adjusted for age, sex, PS and tumour stage, tumour grade did not remain predictive of OS or PFS when adjusted in the multivariable analysis.

The proportional hazards assumption was met and linearity of the Ki67 variable was confirmed. The combined tumour grade and continuous Ki67 model was tested for interaction terms, which did not improve performance. The model underwent internal validation with 500 bootstrapped samples, which suggested underfitting of the model, with <10% change in the calibration slope (from 1 to 0.93, 95% CI 0.48–1.79) and <1% change in the c-statistic.

Four clinical groups were generated, by combining Ki67 (as a binary variable) with tumour grade: Group 1 (Ki67<30%, low tumour grade), group 2 (Ki67<30%, high tumour grade), group 3 (Ki67≥30%, low tumour grade) and group 4 (Ki67≥30%, high tumour grade), with separation of survival in group 4 compared with group 1, and borderline separation of group 3 compared with group 1 (table 2). Median survival for group 1 was 660.5 days (IQR 329–1297), group 2 507.5 days (315–890), group 3 332 days (162.5–530.5) and group 4 300 days (124–366).

**TABLE 2:** Prognostic survival models for Ki67, tumour grade, and Ki67 in combination with tumour grade for overall survival (OS) (primary outcome) and progression-free survival (PFS) (secondary outcome), unadjusted and adjusted for age, sex, WHO performance status and tumour stage

	n	Primary outcome: OS		Secondary outcome: PFS	
		Crude HR (95% CI)	Adjusted HR (95% CI)	Crude HR (95% CI)	Adjusted HR (95% CI)
**Ki67% (continuous)**	97	1.02 (1.01–1.03), p<0.001	1.02 (1.01,1.03), p=0.001	1.02 (1.01–1.02), p=0.001	1.01 (1.00–1.03), p=0.018
**Ki67 at 30% cut-off**	97	2.37 (1.51–3.71), p<0.001	2.63 (1.53–4.49), p<0.001	2.09 (1.35–3.23), p=0.001	1.94 (1.17–3.20), p=0.010
**Two-tier tumour grade**	98	1.83 (1.13–2.97), p=0.014	1.55 (0.89–2.70), p=0.125	1.70 (1.08–2.66), p=0.021	1.66 (1.00–2.78), p=0.055
**Group 1: Ki67<30%, low grade**	46	1 (reference)	1 (reference)	1 (reference)	1 (reference)
**Group 2: Ki67<30%, high grade**	10	0.73 (0.26–2.05), p=0.543	0.64 (0.22–1.86), p=0.413	0.99 (0.42–2.35), p=0.983	1.14 (0.46–2.78), p=0.780
**Group 3: Ki67≥30%, low grade**	20	1.78 (1.00–3.15), p=0.049	2.01 (0.99–4.10), p=0.054	1.70 (0.98–2.96), p=0.061	1.64 (0.85–3.15), p=0.138
**Group 4: Ki67≥30%, high grade**	21	3.70 (2.09–6.55), p<0.001	3.48 (1.79–6.75), p<0.001	2.75 (1.60–4.71), p<0.001	2.42 (1.31–4.48), p=0.005

### Subgroup analysis: stratification by tumour grade

**FIGURE 3:**
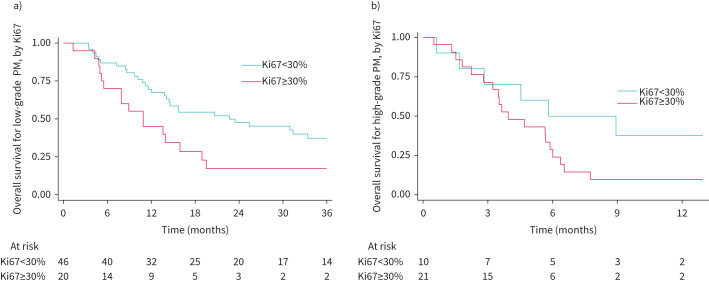
Survival for patients with low and high tumour grade, stratified by Ki67% at diagnosis. PM: pleural mesothelioma.

For those with high-grade PM (n=31), Ki67, as a continuous variable, predicted OS (HR 1.03, 1.01–1.06, adj HR 1.04, 1.01–1.07), and when dichotomised at 30% (HR 3.90, 1.42–10.72, adj HR 7.00, 1.71–28.7). For those with low grade PM (n=66), Ki67, as a continuous variable, predicted OS (HR 1.01, 1.0–1.02, adj HR 1.02, 1.00–1.04) and when dichotomised at 30% (HR 1.74, 0.98–3.09, adj HR 2.44, 1.14–5.23). Survival curves are shown in figure 3. The predictive relationship for PFS in this subgroup analysis was not maintained.

### Sensitivity analysis: treatment status

Ki67 (as a continuous variable and dichotomised at 30%) was predictive of OS regardless of treatment status (table 3).

**TABLE 3:** Stratified by treatment status, overall survival (OS) for tumour grade with and without the addition of Ki67, unadjusted and adjusted for age, sex, WHO performance status and tumour stage

	Received treatment, n=47		No treatment received, n=39	
	Crude HR (95% CI)	Adjusted HR (95% CI)	Crude HR (95% CI)	Adjusted HR (95% CI)
**Ki67 (continuous)**	1.02 (1.00–1.03), p=0.021	1.02 (1.01–1.04), p=0.012	1.03 (1.01–1.04), p=0.001	1.04 (1.02–1.06), p=0.001
**Ki67 (dichotomised at 30%)**	2.07(1.07–4.00), p=0.031	3.11 (1.35–7.15), p=0.008	3.99 (1.77–9.01), p=0.001	4.21 (1.39–12.75), p=0.011
**Two-tier tumour grade**	2.32 (1.07–5.04), p=0.033	1.94 (0.78–4.80), p=0.153	1.47 (0.71–3.01), p=0.299	1.50 (0.60–3.74), p=0.382
**Group 1: Ki67<30%, low grade**	1 (reference)	1	1 (reference)	1 (reference)
**Group 2: Ki67<30%, high grade**	0.57 (0.08–4.38), p=0.593	0.81 (0.11–7.23), p=0.898	0.91 (0.25–3.26), p=0.880	0.87 (0.21–3.57), p=0.848
**Group 3: Ki67≥30%, low grade**	1.42 (0.63–3.24), p=0.400	2.88 (1.04–8.00), p=0.042	4.25 (1.54–11.72), p=0.005	5.22 (1.16–23.6), p=0.032
**Group 4: Ki67≥30%, high grade**	4.55 (1.81–11.44), p=0.001	4.02 (1.39–11.60), p=0.010	4.86 (1.83–12.9), p=0.001	5.75 (1.59–20.85), p=0.008

The prognostic models combining tumour grade with Ki67 (continuous and dichotomised at 30%), also predicted OS (table 3). In those receiving treatment, Harrell's c-statistic for OS increased from 0.56, 95% CI 0.50–0.62 for tumour grade alone to 0.64, 0.57–0.70 when combined with Ki67. In those not receiving treatment, Harrell's c-statistic increased from 0.57, 0.51–0.63 for tumour grade alone to 0.68, 0.61–0.76 when combined with Ki67.

When grouped by treatment status, Ki67 predicted PFS in those who received treatment (HR 1.02, 1.00–1.04, adj HR 1.02, 1.00–1.04) but not for those who were untreated (HR 1.01, 1.00–1.02, adj HR 1.01, 1.00–1.03).

### Exploratory analysis: individual parameter analysis

Three-tier nuclear grade, necrosis, severe nuclear atypia, mitotic score, NLR and albumin all predicted OS in univariate analysis and when adjusted for age, sex and PS. Necrosis, TLS and severe nuclear atypia also predicted PFS in crude and adjusted models (table 4). Ki67 dichotomised at 30% positively correlated with two- and three-tier nuclear grade, necrosis and mitotic activity (p<0.001) (appendix H, supplementary material).

**TABLE 4:** Survival models (crude and adjusted for age, sex, performance status (PS) and TNM tumour stage) for overall survival (OS) and progression-free survival (PFS) for individual pre-specified variables, unadjusted and adjusted for age, sex, WHO PS and tumour stage

Histological variable	n	Primary outcome: OS		Secondary outcome: PFS	
		Crude HR	Adj PS, sex, age, TNM	Crude HR	Adjusted for PS, sex, age, TNM
**Three-tier nuclear grade**	98	1: reference; 2: 1.92 (1.17–3.17), p=0.01; 3: 3.43 (1.53–7.68), p=0.003	1; 2: 1.77 (1.01–3.08), p=0.045; 3: 3.56 (1.46–8.69), p=0.005	1; 2: 1.40 (0.89–2.20), p=0.151; 3: 1.69 (0.79–3.62), p=0.177	1; 2: 1.19 (0.69–2.08), p=0.529; 3: 1.55 (0.60–4.04), p=0.365
**Necrosis**	98	1.88 (1.16-.3.04), p=0.011	1.82 (1.09–3.02), p=0.021	1.43 (0.91–2.24), p=0.118	1.46 (0.86–2.50), p=0.164
**Tertiary lymphoid structures**	98	0.67 (0.41–1.01), p=0.100	0.69 (0.39–1.21), p=0.191	0.60 (0.38–0.94), p=0.026	0.52 (0.31–0.88), p=0.014
**Nuclear atypia**	99	Mild: 1 (reference); mod: 1.63 (0.92–2.89), p=0.092; severe: 3.68 (1.80–7.54), p<0.001	Mild: 1; mod: 1.77 (0.93–3.38), p=0.084; severe: 4.38 (1.82–10.56), p=0.001	Mild: 1; mod: 1.35 (0.80–2.27), p=0.256; severe: 2.32 (1.20–4.47), p=0.012	Mild: 1; mod: 1.67 (0.77–2.44), p=0.291; severe: 2.43 (1.08–5.44), p=0.031
**Mitotic score**	98	1: reference; 2: 2.31 (1.33–4.01), p=0.003; 3: 1.94 (1.45–3.30), p=0.014	1; 2: 1.97 (0.96–4.04), p=0.065; 3: 1.98 (0.92–4.27), p=0.082	1; 2: 1.59 (0.95–2.66), p=0.075; 3: 1.56 (0.96–2.66), p=0.076	1; 2: 0.82 (0.44–1.52), p=0.532; 3: 0.94 (0.49–1.81), p=0.849
**Host response**	98	Mild: 1 (reference); mod: 0.76 (0.46–1.56), p=0.284; intense: 1.08 (0.57–2.22), p=0.828	Mild: 1; mod: 0.56 (0.30–1.04), p=0.068; intense: 1.09 (0.47–2.55), p=0.841	Mild: 1; mod: 0.67 (0.42–1.01), p=0.091; intense: 0.52 (0.26–1.01), p=0.072	Mild: 1; mod: 0.63 (0.36–1.09), p=0.096; intense: 0.50 (0.21–1.14), p=0.099
**BAP1**	98	1.54 (0.96–2.48), p=0.076	1.34 (0.78–2.32), p=0.292	1.0 (0.64–1.55), p=0.993	0.97 (0.58–1.61), p=0.904
**NLR**	95	1.08 (1.04–1.13), p<0.001	1.06 (1.01–1.12), p=0.015	1.04 (1.0–1.08), p=0.079	1.02 (0.97–1.07), p=0.453
**Albumin**	90	0.96 (0.93–1.0), p=0.033	1.00 (0.95–1.05), p=0.949	1.00 (0.96–1.05), p=0.820	1.05 (0.99–1.11), p=0.104

HR: hazard ratio; NLR: neutrophil–lymphocyte ratio.

### Exploratory analysis: alternative models

Two alternative models were considered (appendix I, supplementary material): one in which Ki67 was combined with the individual components of tumour grade and a second which included Ki67 as the only marker of proliferation (*i.e.* without mitotic count) alongside the nonproliferative components of tumour grade (necrosis and nuclear atypia). Discriminative ability for OS was very similar in the models with and without mitotic score (Harrell's c-statistic 0.66 *versus* 0.65, respectively) and likelihood ratio testing suggested there was little to differentiate between the models statistically (p=0.9255).

## Discussion

This is the largest study to date analysing Ki67 in epithelioid PM patients who underwent predominantly nonsurgical treatment [24].

We have shown that tumour grade, the current WHO standard, is augmented by Ki67 staining: a cost-effective, simple IHC test, analysed *via* microscopy, without the need for digital image analysis (DIA). Reporting Ki67, dichotomised at 30%, alongside tumour grade also predicted PFS. This information could support patients’ and clinicians’ decision-making around treatment.

Our internally validated findings confirm previous work showing Ki67 predicts survival and correlates with tumour grade [14].

We further validate the two- and three-grade system [13, 25, 26], both developed in predominantly surgical cohorts. Interobserver agreement for tumour grade was higher than in previous studies (Cohen's kappa 0.67 *versus* 0.47 [14] and 0.28 [27]). This likely reflects increased pathologist experience rather than international variability.

Ki67 was first identified as a prognostic marker in PM in 1998 [22] and has since shown promise in distinguishing long- from short-surviving patients [16, 17, 28, 29] and in predicting OS and PFS [30, 31]. This study confirms the relationship in a nonsurgical population.

There has been little agreement on optimal dichotomisation, with cut-offs from 10–30%, often chosen at the sample median. This study validates a 30% cut-off, the value originally used in 1998 [22] and chosen by Galeano *et al.* [14]. Ki67 split at 30% was also the most reproducible marker, making it an excellent candidate for clinical use. We propose ≥30% as the optimal cut-off for outcome prediction in PM.

We used a standard visual counting technique for assessing Ki67, which is straightforward and takes a few minutes per case. While Galeano *et al.* [14] showed DIA as an alternative, we found good reproducibility using the visual method. Lack of DIA should therefore not hinder routine Ki67 reporting.

Limitations include omission of treatment status from prediction models, which may have been influenced by tumour grade. However, most of this cohort were diagnosed before its introduction in 2021 [32], mitigating its impact. Despite the introduction of dual immunotherapy during the study period, the effect on outcomes will have been minimised by the low number of patients receiving immunotherapy in our cohort and the limited survival benefit of immunotherapy over chemotherapy in epithelioid PM. Additionally, sensitivity analysis showed the model was predictive of OS regardless of treatment status, although the predictive power for PFS was impacted. This may in part reflect the inherent limitations of CT scans, in that disease progression requires a clinical CT scan to be recognised. Variability in follow–up intervals could theoretically introduce informative censoring, but as only a small number of patients were censored without progression or death, the potential bias in our PFS estimates is minimal.

The 30% threshold was derived and tested within the same dataset, which may introduce optimism. While this can limit generalisability, the threshold was selected based on HRs and model fit across multiple candidate values and its plausibility is supported by prior work that has independently identified the same cut-off [14]. When four groups were generated from Ki67 (30% cut-off) and tumour grade, low Ki67 did not separate by tumour grade. This might be explained by low numbers in the low-Ki67, high-grade group but further work in a larger cohort could be useful to explore this further. However, Ki67 effectively stratified each tumour grade into distinct risk groups, providing valuable additional information at diagnosis. This could enable clinicians to identify the highest risk patients to determine who may benefit from earlier intervention.

The tumour grading system was introduced following evaluation of several scoring systems that use various histological parameters. Pelosi *et al.* [33] used interobserver agreement to select Ki67, necrosis, mitotic count and histological subtype. As with our system, Pelosi *et al.* included two indices of proliferation, arguing that using both reduced errors reported in other validated systems [34]. We have shown that a prognostic model using Ki67 and all components of tumour grade discriminates well, with little difference when mitotic score is excluded. Discrimination for the combined score is lower than that reported by Brims *et al.* [35] for their decision tree development cohort, and while it is comparable with the c-index in the validation cohort of Brims *et al.,* it remains moderate. Although adding Ki67 to tumour grade leverages the already implemented and validated grading system, the combination of Ki67 and tumour grade would also require external validation before introduction into clinical practice [11].

Although this analysis adjusted for tumour stage, it was not included in the original tumour grade validation study and is weaker survival predictor [13, 26]. Stage also plays little role in treatment decisions particularly as most cases of PM are nonoperable [23]. However, the new TNM9 staging system, including composite tumour thickness measurement, shows good separation between prognostic groups [36, 37], although interobserver reproducibility is unreported. A combined radiological–pathological prognostic score with TNM9 and our proposed system could further improve prognostic accuracy.

In summary, Ki67 performs well as an independent outcome predictor in epithelioid PM and outperforms tumour grade in predicting OS and PFS. When combined, tumour grade and Ki67 outperform tumour grade alone and Ki67 further stratifies each tumour grade. This scoring systems is reliable across age, tumour stage, PS and treatment status.

Supported by our validated data, tumour grade plus Ki67 dichotomised at 30% should be considered as a new reporting standard in epithelioid PM.
